# Perivascular Delivery of Notch 1 siRNA Inhibits Injury-Induced Arterial Remodeling

**DOI:** 10.1371/journal.pone.0084122

**Published:** 2014-01-08

**Authors:** Eileen M. Redmond, Weimin Liu, Katie Hamm, Ekaterina Hatch, Paul A. Cahill, David Morrow

**Affiliations:** 1 Department of Surgery, University of Rochester Medical Center, Rochester, New York, United States of America; 2 Vascular Health Research Centre, Dublin City University, Dublin, Ireland; Institut National de la Santé et de la Recherche Médicale, France

## Abstract

**Objectives:**

To determine the efficacy of perivascular delivery of Notch 1 siRNA in preventing injury-induced arterial remodeling.

**Methods and Results:**

Carotid artery ligation was performed to induce arterial remodeling. After 14 days, morphometric analysis confirmed increased vSMC growth and subsequent media thickening and neointimal formation. Laser capture microdissection, quantitative qRT-PCR and immunoblot analysis of medial tissue revealed a significant increase in Notch1 receptor and notch target gene, Hrt 1 and 2 expression in the injured vessels. Perivascular delivery of Notch 1 siRNA by pluronic gel inhibited the injury-induced increase in Notch 1 receptor and target gene expression when compared to scrambled siRNA controls while concomitantly reducing media thickening and neointimal formation to pre-injury, sham-operated levels. Selective Notch 1 knockdown also reversed the injury-induced inhibition of pro-apoptotic Bax expression while decreasing injury-induced anti-apoptotic Bcl-X_L_ expression to sham-operated control levels. In parallel experiments, proliferative cyclin levels, as measured by PCNA expression, were reversed to sham-operated control levels following selective Notch 1 knockdown.

**Conclusion:**

These results suggest that injury-induced arterial remodeling can be successfully inhibited by localized perivascular delivery of Notch 1 siRNA.

## Introduction

Atherosclerosis and arterial occlusion as a result of flow-limiting stenosis is a leading cause of myocardial infarction and sudden death [34] [26]. The arterial remodeling responsible for atherosclerosis is characterized by a vascular pathology where medial thickening, neointimal formation and subsequent narrowing of the lumen are the predominant features [26] [27] [1]. This remodeling can be outward and expansive or inward and constrictive and is also characteristic of restenosis following balloon angioplasty and in transplant vasculopathy [5]. Changes in vascular smooth muscle cell (vSMC) growth and survival play an important role in medial thickening and neointimal formation during this arterial remodeling in response to injury, however the mechanisms remain unclear [15] [11]. As similar changes are also apparent during vasculogenesis and embryonic development [8] [4], we and others have postulated that the control of vSMC growth and subsequent vascular remodeling in disease states and following injury may share similar signaling pathways.

Notch signaling plays a pivotal role in the differentiation and function of adult vSMCs, whose growth and migration are key processes in the pathophysiology of arterial remodeling [29] [20] [33]. Several groups, including our own, have described a role for Notch signaling *in vitro* and *in vivo* in repressing vSMC differentiation, an effect that is mediated via the induction of its target genes hairy enhancer of split [HES] and *HRT* [20] [31] [32]. Conversely a number of gene inactivation studies in mice have clearly demonstrated that Notch signaling promotes vSMC differentiation during development [7] [9]. Moreover, Notch signaling has been shown to promote the differentiation of bone marrow-derived cells in to SMC-like cells resulting in arterial lesion formation [6].

We have recently shown that in the carotid ligation mouse model, there is increased expression of several components of the Notch pathway, including Notch 1 and 3, Jagged 1 and 2, and Notch target genes Hrt 1 and 2 [19] [25]. This and other studies highlight Notch as a critical factor in determining the vSMC proliferative response to arterial injury [19] [30]. Recent evidence points to a preferential role for Notch 1, rather than Notch 3, in mediating medial thickness and neointimal formation after vascular injury through regulation of its downstream target genes hrt-1 and 2 [13]. Although further elucidation of the role of Notch 1 in the remodeling response is clearly warranted, no study to date has directly demonstrated the functional repercussion of local Notch 1 inhibition using novel delivery systems as a foundation for targeted therapies. Given these reports in the literature and our previous studies supporting a role for Notch signaling components in vSMC *in vitro, and in vivo* in the injury-induced remodeled vessel, the current study examined whether local perivascular delivery of Notch1 siRNA could inhibit the vSMC growth and reverse subsequent medial thickening and neointimal formation, both of which are hallmarks of injury-induced arterial remodeling.

## Methods

### Mouse Carotid Artery Partial Ligation

The carotid artery ligation model of vascular injury and remodeling was performed essentially as described utilizing 6-8 week male C57BL/6 mice [10] [16]. All procedures were approved by the University of Rochester Animal Care Committee. After buprenorphine analgesia and induction of anesthesia using inhalational isoflurane, the mouse was positioned on a clean operating table, with a warming pad to maintain body temperature. The animal was clipped and the surgical site prepped using betadine solution and alcohol. A midline cervical incision was made. With the aid of a dissecting microscope, the left external and internal carotid arterial branches were isolated and ligated with 6-0 silk suture reducing left carotid blood flow to flow via the patent occipital artery. The neck incision (2 layers, muscle and skin) was closed with a running suture using 4-0 coated Vicryl. Partial ligation of the left carotid artery in this manner resulted in a decrease in blood flow in the left carotid artery, concomitant with an increase in the right carotid artery, with an intact endothelial monolayer, when compared to sham-operated control.

### siRNA delivery *in vivo*

Select *in vivo* ready siRNAs for Notch 1(NM_008714.3), (Cat. # 4457308, Life Technologies, Grand Island, NY) were pre mixed with lipofectamine (Life Technologies, Grand Island) based transfection reagents and optimum (Invitrogen) to a total volume of 30 µl. This was then mixed with 40 µl of pluronic gel. Pluronic gel solutions (Sigma, St. Louis, MO) at 1 mg/ml were prepared and kept at 4°C. Following carotid ligation, this 70 µl solution was applied to the carotid artery. On contact with tissues at 39°C the pluronic gel solidifies instantaneously, generating a translucent layer that envelops the treated region. The wound was then closed immediately after the application of the gel. Treated vessels were removed at 14 days post ligation for analysis. A high transfection efficiency (70%) was achieved with this method as evident by visualization of Alexa Fluor-tagged control siRNA (A kind gift from Nitin Puri, Life Technologies, Grand Island, NY) showing localized delivery throughout the vessel [25].

### Preparation of Carotid Artery RNA

Mice were perfused with heparin/saline solution. Carotid arteries were collected in Trizol (Invitrogen) and homogenized using an Ultra-Turrax tissue disperser and RNA was prepared according to the manufacturers specifications.

### Western Blot Analysis

Proteins from vessels (12–15 µg) were resolved on SDS-PAGE (12% resolving, 5% stacking) prior to transfer onto nitrocellulose membrane (Amersham Biosciences, Piscataway, NJ). Membranes were stained with Ponceau S and probed for Glyceraldehyde 3-phosphate dehydrogenase (GAPDH) to ensure equal protein loading and transfer and rinsed in wash buffer (PBS containing 0.05% Tween-20) before being probed as described previously [29]. Antibodies were purchased from Abcam for Notch 1 (ab52627), Hrt-1 (ab22614), Hrt-2 (ab25404), Bax (ab7977), Bcl-X_L_ (ab32370) and PCNA (ab29).

### Quantitative real-time RT-PCR

Total RNA (0.5-1 µg), isolated from vessels using Qiagen RNeasy kit (Valencia, CA) was reverse-transcribed using iscript™ cDNA Synthesis kit from BIO-RAD (Carlsbad, CA). The gene-specific oligonucleotide sequences were as previously described [16]. Real-Time RT-PCR was performed using the Stratagene MX3005 machine and the SYBER green jumpstart PCR kit (Sigma, St. Louis, MO) as described by the manufacturer.

### Immunohistochemistry and Histomorphometry

Mice were perfusion fixed with 10% paraformaldehyde in sodium phosphate buffer (pH 7.0), 14 days post ligation, and the carotids harvested and embedded in paraffin. Starting 500 µM below the carotid bifurcation landmark, a series of cross-sections (10×5 µm) were made, every 200 µm through 2 mm length of carotid artery. Cross-sections were stained with Verhoeff-Van Gieson stain for elastic laminae and sections were imaged using a Nikon TE300 microscope equipped with a Spot RT digital camera (Diagnostic Instruments). Digitized images were analyzed using MCID image software. Assuming a circular structure *in vivo*, the circumference of the lumen was used to calculate the lumen area, the intimal area was defined by the luminal surface and internal elastic lamina (IEL), the medial area was defined by the IEL and external elastic lamina (EEL) and the adventitial area was the area between the EEL and the outer edge, essentially as described previously [10]. All histomorphometric analysis was performed “blinded” by the same observer.

### Confocal Immunohistochemistry

Immunofluorescence for smooth muscle actin (Sigma; A2547), activated Notch 1 (NICD; Abcam, ab8925) and dual smooth muscle actin/Notch1 was performed on sham and ligated sections as previously described [30].

### Data Analysis

Results are expressed as mean ± SEM. Experimental points were performed in triplicate, with a minimum of 3 independent experiments (VSMC), or a minimum of 5 animals per group. An ANOVA test was performed on cell count data and a Wilcoxon Signed rank test was used for comparison of two groups when compared to normalized control. A value of p≤0.05 was considered significant.

## Results

### Perivascular delivery of Notch1 siRNA in Injured Vessel inhibits Notch Signalling Component Expression

**Figure 1:**
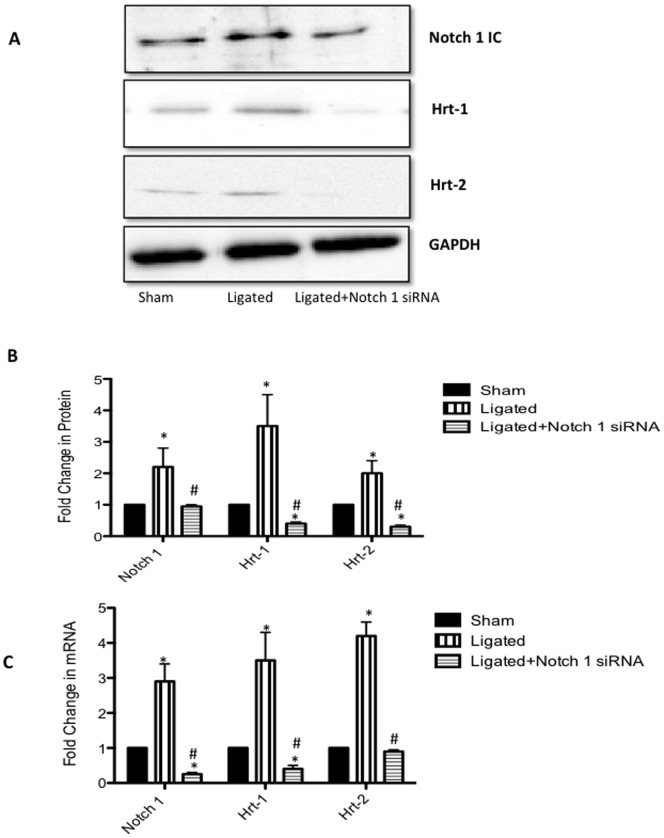
Perivascular delivery of Notch 1 siRNA in Injured Vessel inhibits Notch Signaling Component Expression. (A) Representative Western Blot and (B) cumulative protein data for Notch 1 IC and Notch target gene (Hrt-1 and Hrt-2) expression 14 days after carotid ligation in sham, ligated (scrambled siRNA) and ligated+Notch 1 siRNA vessels. (C) qRT-PCR analysis of Notch 1, Hrt-1 and Hrt-2 mRNA levels 14 days after carotid ligation in sham, ligated (scrambled siRNA) and ligated+Notch 1 siRNA vessels. Data are normalized to GAPDH and represent the mean ± SEM, n = 6.

**Figure 2:**
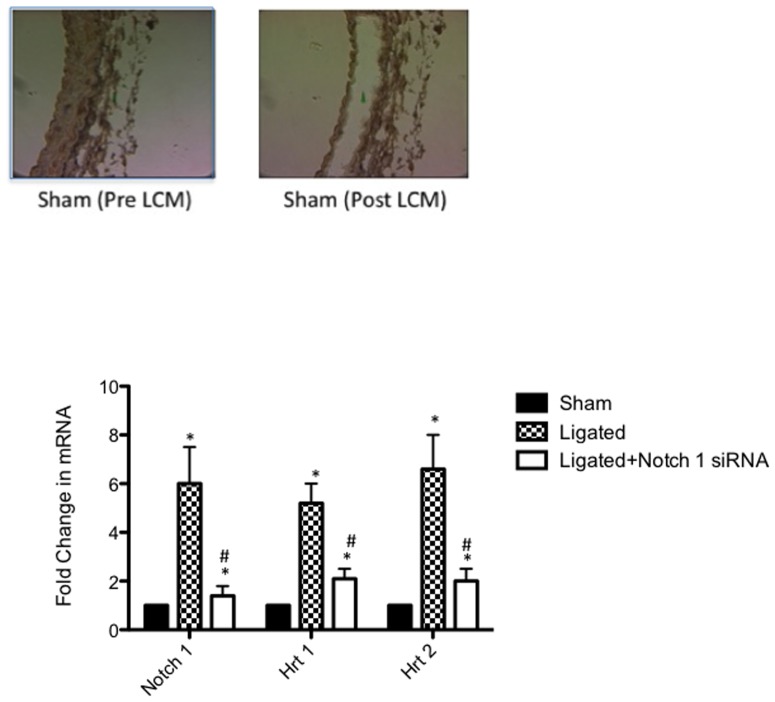
Perivascular delivery of Notch 1 siRNA inhibits Medial SMC Notch Signaling Component Expression. (A) Representative image of medial SMC layer dissected by Laser Capture Microdissection (LCM) for mRNA analysis. (B) qRT-PCR analysis of medial SMC Notch 1, Hrt-1 and Hrt-2 mRNA levels 14 days after carotid ligation in sham, ligated (scrambled siRNA) and Ligated+Notch 1 siRNA vessels. Data are normalized to GAPDH and represent the mean ± SEM, n = 6.

Notch component mRNA and protein levels were determined in the carotid arteries of sham, scrambled siRNA ligated (ligated) and Notch1 siRNA ligated vessels. There was a significant increase in Notch 1 and Hrt-1 and -2 protein expression (Figure 1A and B) and mRNA levels (Figure 1 C) in ligated vessels when compared to sham. The injury-induced increases in Notch 1 protein (Figure 1 A, B) and mRNA levels (Figure 1 C) were reversed following selective localized knockdown of Notch 1 by siRNA when compared to ligated vessels treated with control scrambled siRNA. Notch 1 IC protein levels were inhibited by perivascular delivery of Notch 1 siRNA to levels comparable to γ-secretase inhibition of Notch 1 IC Expression by DAPT (20 µM, 24 h), (Figure S2 in File S1). Similarly, injury-induced increases in Hrt-1 and Hrt-2 protein (Figure 1 A, B) and mRNA expression (Figure 1 C) were significantly attenuated following localized Notch1 gene knockdown. The effect of localized inhibition of the Notch 1 receptor using targeted siRNA on medial vSMC Notch and Notch target gene expression (Hrt-1, Hrt-2) was determined utilizing laser capture microdisection (LCM) to microdissect the tunica media in the injury-induced remodeled artery. As in whole vessel analysis, ligation injury-induced increase in medial vSMC Notch1 mRNA levels was reversed following selective knockdown of Notch 1 by siRNA. Similarly, the injury-induced increase in medial vSMC Hrt-1 and Hrt-2 mRNA expression was significantly attenuated following localized Notch1 gene knockdown (Figure 2). In addition, cytoplasmic staining of Notch 1 IC in medial/intimal vSMC in the ligated carotid artery was also determined by dual Notch 1/DAPI staining by confocal immunofluorescence (Figure S3 in File S1).

### Localized Notch1 inhibition attenuates injury-induced increases in Vascular Cell Growth

**Figure 3:**
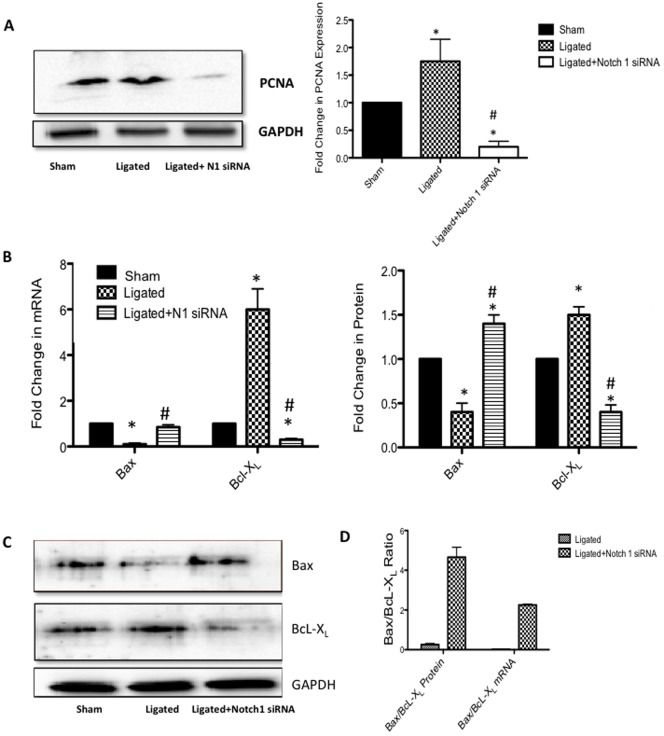
Localized Notch 1 inhibition attenuates injury-induced increases in Vascular Cell Growth. (A) Representative Western Blot and cumulative data of proliferating cell nuclear antigen (PCNA) protein expression (B) qRT-PCR analysis of pro-apoptotic Bax and anti-apoptotic BcL-X_L_ (C) Representative Western Blot and cumulative data of pro-apoptotic Bax and anti-apoptotic BcL-X_L_ and (D) Bax/Bcl-X_L_ ratios 14 days after carotid ligation in sham, ligated (scrambled siRNA) and ligated+Notch 1 siRNA vessels. Data are normalized to GAPDH and represent the mean ± SEM, n = 6.

The effect of localized delivery of Notch 1 siRNA on ligation injury-induced vSMC growth was also assessed. vSMC proliferation as determined by examining the expression of the cyclin, PCNA, was significantly attenuated following selective knockdown of Notch 1 by siRNA, when compared to the scrambled ligated control (Figure 3 A). Similarly, injury-induced decreases in pro-apoptotic Bax mRNA (Figure 3 B) and protein expression (Figure 3 C) were significantly attenuated following Notch1 gene knockdown. In parallel studies, injury induced increases in anti-apoptotic Bcl-X_L_ mRNA (Figure 3 B) and protein expression (Figure 3 C) were significantly attenuated following Notch1 gene knockdown when compared to the scrambled ligated controls. Overall, localized Notch 1 Inhibition with targeted siRNA resulted in a significant decrease in proliferation concomitant with a shift in the BAX:Bcl-X_L_ ratio towards cell death (Figure 3D).

### Perivascular delivery of Notch1 siRNA prevents injury-induced Vascular Remodeling

**Figure 4:**
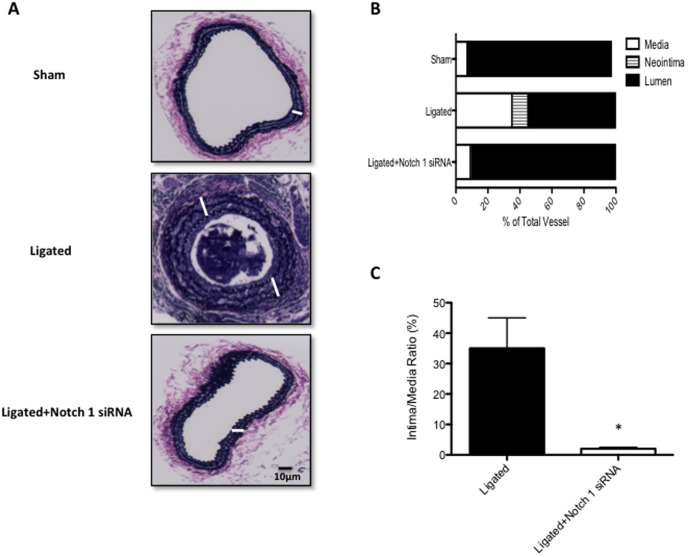
Perivascular delivery of Notch 1 siRNA prevents injury-induced Vascular Remodeling. (A) Representative Verhoeff-van Gieson staining of carotid artery from C57BI6/J mice 14 days after ligation in the absence or presence of scrambled siRNA (ligated) or Ligated+Notch 1 siRNA. White bars mark media area from internal elastic lamina to the external elastic lamina. (B) Carotid artery vessel media, neointima and lumen volumes (evaluated over a 1 mm carotid length) for sham, ligated and Ligated+Notch 1 siRNA groups. (C) Carotid artery Intima/Media ratios for ligated and Ligated+Notch 1 siRNA groups. Data are mean ± SEM, 4 sections analyzed/animal, n = 6 animals for each group.

**Figure 5:**
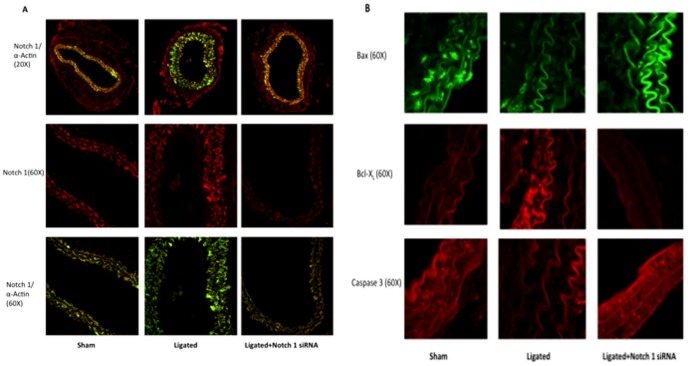
Perivascular delivery of Notch 1 siRNA prevents injury-induced Vascular Remodeling. (A) Photomicrographs of confocal immunofluorescence staining for Notch 1, SMC α-actin or dual staining for α-actin/Notch 1 in carotid arteries 14 d after ligation in sham, ligated (scrambled siRNA) and ligated+Notch 1 siRNA vessels. (B) Photomicrographs of confocal immunofluorescence (60X) staining for Bax, Bcl-X_L_ and Caspase-3 expression in carotid arteries 14 d after ligation in sham, ligated control (scrambled siRNA) and ligated Notch 1 siRNA vessels. Data are mean ± SEM, 4 sections analyzed/animal, n = 6 animals for each group.

Partial ligation of the carotid artery in a wildtype C57BL/6J mouse resulted in a 90% decrease in blood flow and induced a reproducible remodeling response, assessed 2 weeks post ligation, that included neointimal lesion formation and an increase in SMC medial growth as compared to sham-operated vessels (Figure 4 A). This remodeling response was markedly reduced in mice that received perivascular delivery of Notch 1 siRNA immediately post-ligation, when compared to scrambled siRNA controls. The injury-induced increase in media thickening (white bars marking media area between internal elastic lamina and external elastic lamina) and neointimal formation was reduced to sham-operated levels following localized Notch1 gene knockdown (Figure 4 A, B), while the injury-induced decreases in lumen volume were also abrogated to sham-operated control levels (Figure 4 A, B). The intima/media ratios for injured carotid arteries were markedly reduced following localized Notch 1 knockdown (Figure 4 C). Confocal immunofluorescence examination of fixed tissue sections of carotids from sham operated control vessels, ligated scrambled siRNA vessels and Notch 1 siRNA ligated vessels indicated high levels of Notch 1 predominantly in the SMC rich media as evidenced by co-staining with SMC α-actin. Injury-induced expression levels of Notch1 were reduced to sham-operated levels following localized inhibition of Notch1 (Figure 5. A). In parallel experiments, dual PCNA/α-Actin expression was determined in injury-induced remodeled vessels. Carotid artery ligation increased SMC PCNA expression in the remodeled vessel, an increase that was inhibited to sham-operated control levels following perivascular delivery of Notch 1 siRNA (Figure S1 in File S1). In addition, injury-induced expression of pro-apoptotic Bax was significantly reduced while anti-apoptotic BcL-X_L_ concurrently increased when compared to sham-operated levels (Figure 5.B). Perivascular delivery of Notch 1 siRNA in ligated vessels maintained the Bax and Bcl-X_L_ levels at sham-operated control levels. Similarly to pro-apoptotic Bax, carotid artery ligation decreased caspase-3 expression while perivascular delivery of Notch 1 siRNA in ligated vessels maintained Caspase 3 expression to sham-operated control levels (Figure 5.B).

## Discussion

Intimal medial thickening and restenosis have a limiting effect on the success of angioplasty and bypass surgery due to the pathological growth of vSMCs in treated vessels [14] [3]. As a result, a greater understanding of the signaling pathways that arbitrate these changes in vSMC growth remain key to advancing our knowledge of the etiology of atherosclerosis, arteriosclerosis, vascular rejection, venous graft restenosis and coronary intervention failure, all of which are characterized by increased vSMC growth and intimal medial thickening. We and others have addressed the specific role of the Notch signaling pathway in regulating vSMC phenotype changes characteristic to intimal medial thickening in the injured artery [18] [17] [22] [32]. This is mainly due to a paradigm suggesting that developmental gene regulatory networks are often recapitulated in adult vascular cells in the context of phenotypic modulation, vascular remodeling, and repair, all of which are hallmarks of adult vascular disease. The clinical importance and therapeutic potential of modulating vascular phenotype in the injured artery remains abundantly clear. In addition to increasing our knowledge of the specific gene networks involved in this process and subsequent development of inhibitory agents that reduce this pathogenic process, the technical challenge associated with their therapeutic application also represents an equally demanding endeavor. The novelty of our current study is that for the first time we show that localized delivery of a targeted Notch 1 siRNA competently inhibits injury-induced vSMC growth and vessel remodeling.

We established that localized inhibition of Notch1 in the injury-induced remodeled artery following perivascular pluronic gel delivery of Notch 1 siRNA markedly inhibited the well-characterized medial thickening and neointimal formation that causes the lumen narrowing associated with this model. The flow reduction carotid artery ligation model in C57BL/6J mice as previously reported by us and others results in significant medial thickening and neointimal formation and subsequent lumen narrowing 14 days post ligation concomitant with a selective increase in Notch signaling components [10] [16] [17]. Our previous studies report a preferential increase in the expression of the Notch 1 versus the Notch 3 receptor [16], [25]. Here we show that this increase in Notch1 receptor expression is predominantly localized in vSMC as evident by co-staining with alpha actin. In addition, Laser Capture Microdissection (LCM) was utilized to remove the SMC rich medial layer, which was then analyzed for Notch/Hrt RNA expression. The data in parallel with whole vessel analysis confirmed that injury–induced vascular remodeling is accompanied by increased Notch 1/Hrt signaling within medial vSMC which results in a pro-proliferative phenotype in these injured vessels promoting vessel remodeling which could be inhibited to sham-operated control levels with Notch1 gene knockdown.

In this study, we adopted a perivascular delivery system of gene specific siRNAs utilizing a pluronic gel [25] [28]. Following pluronic gel delivery of Notch 1 siRNA, which targets the desired siRNA to an area below the bifurcation of the ligated carotid artery, we achieved a significant inhibition of whole vessel and medial Notch1 mRNA and protein expression, concomitant with decreased SMC medial growth and injury-induced remodeling. Our results compliment recent work by Li *et al* that addressed the role of Notch 1 in neointimal formation following vascular injury [13]. That study utilized heterozygous Notch1 mutants, which indicated a role for Notch1, rather than Notch 3 in mediating SMC growth and neointimal formation after vascular injury through CHF1/Hey2. As mentioned above, we have also previously demonstrated a preferential role for Notch 1 versus Notch 3 following injury-induced remodeling [16]. Here we show for the first time that this injury-induced remodeling can be prevented following localized Notch 1 knockdown using an siRNA platform.

The functional relationship between Notch signaling and vSMC growth *in vitro* and *in vivo* is now well established [29] [2] [32]. In this context, injury induced Notch stimulation results in robust downstream Notch target gene expression within the vessel wall as seen by increased Hrt expression resulting in increased vSMC growth and subsequent remodeling. It is the Hrt's, which feed into the proliferative (e.g. c-myc) and the anti-apoptotic machinery of the cell by regulating both pro-apoptotic Bax and anti-apoptotic Bcl-X_L_ [23]. The balance of proapoptotic Bax and antiapoptotic Bcl-X_L_ is known to be important in determining whether cells die or survive [24]. Our data supports previous studies where anti-apoptotic Bcl-X_L_ is elevated in the injured vessel while pro-apoptotic bax levels are subsequently decreased [24] [21] [17] [35]. Localized knockdown of Notch 1 abrogates the injury-induced changes in these apoptotic markers to sham-operated levels further highlighting a functional relationship between Notch/Hrt signaling and Bax/Bcl-X_L_ expression. We believe this Notch/Hrt regulation of Bax/Bcl-X_L_ signaling to be a key event in the vSMC phenotype switching which drives the injury-induced medial thickening and neointimal formation. This to our knowledge is the first study that directly links Notch1/Hrt and Bax/Bcl-X_L_ signaling together in controlling changes in SMC phenotype resulting from ligation-induced injury.

As the signaling pathways driving injury-induced vessel remodeling are revealed we must be cognizant of the technical challenges that are associated with the therapeutic application of such components that we attempt to inhibit or promote. This study involved a localized delivery system of Notch 1 siRNA, which significantly abrogated the remodeling and vessel stenosis resulting from ligation-induced injury. In our hands, pluronic gel delivery of alexa fluor tagged control siRNA showed high levels of medial expression 7 days post application [25]. The use of siRNAs are becoming among the fastest developing therapeutic approaches for gene and protein inhibition [12]. In addition to their specificity there is also increased interest in their use due to reduced toxicity concerns as a result siRNAs being directly and transiently delivered ex vivo, by pluronic gel or in studies using bypass grafts This in turn greatly reduces the potential for systemic toxicity and also represents a powerful tool in inhibiting the restenosis event, which follows balloon angioplasty for example, by introducing a suitable inhibitor of vessel remodeling [12]. Another therapeutic possibility is to utilize drug-eluting stents to deliver Notch siRNAs to the site of lesions in injured vessels in an attempt to inhibit or reverse injury-induced remodeling. Taking together their specificity and potential for reduced systemic toxicity, it is hoped that this localized treatment of stenosed vessels with Notch1 specific siRNA, could represent a novel therapy for either restenosis which may accompany vein grafting, post angioplasty or atherosclerosis.

## Supporting Information

File S1 **Contains the following:** Figure S1. Perivascular delivery of Notch 1 siRNA modulates PCNA expression in injury-induced vascular remodeling. Photomicrographs of confocal immunofluorescence (30X) staining for dual PCNA (Red)/α-Actin (Green) expression in carotid arteries 14 d after ligation in sham, ligated control (scrambled siRNA) and ligated Notch 1 siRNA vessels. Data are mean ± Sem. Figure S2: γ-secretase inhibition of Notch 1 IC Expression. Representative western blot of Notch 1 IC protein expression in HCASMC following γ-secretase inhibition with 20 µM DAPT for 24 h. Figure S3: Cytoplasmic Staining of Notch 1 IC in Medial/Intimal Smooth Muscle Cells. Photomicrographs of confocal immunofluorescence (30X) for dual staining for Notch 1/DAPI in ligated carotid artery 14 d after ligation. White arrows indicate cytoplasmic staining for Notch 1 IC around DAPI stained nuclei. Data are mean ± SEM, 4 sections analyzed/animal, n = 6 animals for each group.
(PPTX)
